# An environmental assessment and risk map of *Ascaris lumbricoides* and *Necator americanus* distributions in Manufahi District, Timor-Leste

**DOI:** 10.1371/journal.pntd.0005565

**Published:** 2017-05-10

**Authors:** Rebecca Wardell, Archie C. A. Clements, Aparna Lal, David Summers, Stacey Llewellyn, Suzy J. Campbell, James McCarthy, Darren J. Gray, Susana V. Nery

**Affiliations:** 1 Department of Global Health, Research School of Population Health, College of Medicine, Biology and the Environment, The Australian National University, Canberra, ACT, Australia; 2 National Centre for Epidemiology and Population Health, Research School of Population Health, College of Medicine, Biology and the Environment, The Australian National University, Canberra, ACT, Australia; 3 Fenner School of Environment and Society, College of Medicine, Biology and the Environment, The Australian National University, Canberra, ACT, Australia; 4 Clinical Tropical Medicine Laboratory, QIMR Berghofer Medical Research Institute, Herston, Queensland, Australia; 5 School of Public Health, University of Queensland, Brisbane, Australia; 6 Molecular Parasitology Laboratory, QIMR Berghofer Medical Research Institute, Brisbane, Australia; University of Kelaniya, SRI LANKA

## Abstract

**Background:**

In Timor-Leste there have been intermittent and ineffective soil-transmitted helminth (STH) deworming programs since 2004. In a resource-constrained setting, having information on the geographic distribution of STH can aid in prioritising high risk communities for intervention. This study aimed to quantify the environmental risk factors for STH infection and to produce a risk map of STH in Manufahi district, Timor-Leste.

**Methodology/Principal findings:**

Georeferenced cross-sectional data and stool samples were obtained from 2,194 participants in 606 households in 24 villages in the Manufahi District as part of cross sectional surveys done in the context of the “WASH for Worms” randomised controlled trial. Infection status was determined for *Ascaris lumbricoides* and *Necator americanus* using real-time quantitative polymerase chain reaction. Baseline infection data were linked to environmental data obtained for each household. Univariable and multivariable multilevel mixed-effects logistic regression analysis with random effects at the village and household level were conducted, with all models adjusted for age and sex. For *A*. *lumbricoides*, being a school-aged child increased the odds of infection, whilst higher temperatures in the coolest quarter of the year, alkaline soils, clay loam/loam soils and woody savannas around households were associated with decreased infection odds. For *N*. *americanus*, greater precipitation in the driest month, higher average enhanced vegetation index, age and sandy loam soils increased infection odds, whereas being female and living at higher elevations decreased the odds of infection. Predictive risk maps generated for Manufahi based upon these final models highlight the high predicted risk of *N*. *americanus* infection across the district and the more focal nature of *A*. *lumbricoides* infection. The predicted risk of any STH infection is high across the entire district.

**Conclusions/Significance:**

The widespread predicted risk of any STH infection in 6 to 18 year olds provides strong evidence to support strategies for control across the entire geographical area. As few studies include soil texture and pH in their analysis, this study adds to a growing body of evidence suggesting these factors influence STH infection distribution. This study also further supports that *A*. *lumbricoides* prefers acidic soils, highlighting a potential relatively unexplored avenue for control.

**Trial registration:**

ClinicalTrials.gov ACTRN12614000680662.

## Author summary

The majority of soil-transmitted helminth (STH) infections have long-term ramifications on an individual’s health and productivity, contributing to malnutrition, anaemia, and impaired childhood growth. In Timor-Leste there have been intermittent and ineffective STH control programs since 2004. When resources are constrained, having information on the geographic distribution of STH is important to ensure limited resources are targeted to areas most in need. In this study we predicted the risk of *Ascaris lumbricoides*, *Necator americanus* and any STH infection in the Manufahi district of Timor-Leste, providing the first available risk maps for STH infection in any part of the country. To achieve this, we assessed the relationship between selected environmental factors and STH infection to create multivariable models accounting for potential clustering of infections in villages and households. Using these multivariable models, we predicted the risk of *A*. *lumbricoides* and *N*. *americanus* infection across the district based on the environmental variables. We found that the predicted risk of infection with *N*. *americanus* in the age group older than 18 years of age and with any STH in 6–18 year olds was high and widespread across Manufahi, supporting the need for control strategies across the entire geographical region. Our study also highlighted the focal nature of *A*. *lumbricoides* infection and its relationship with soil pH.

## Introduction

Soil-transmitted helminth (STH) infections are predominantly a disease of poverty, typically prevalent in poor tropical and subtropical regions. [1] In 2010, the four main species of human STH were estimated to infect 1.5 billion people worldwide; with whipworm (*Trichuris trichiura*), roundworm (*Ascaris lumbricoides*) and the two main species of hookworm (*Ancylostoma duodenale* and *Necator americanus*) respectively infecting 464.6 million, 819.0 million, and 438.9 million people. [2] The majority of STH infections are insidious for hosts living in impoverished conditions, and have a demonstrable impact on an individual’s health and wellbeing. [1, 3] *A*. *lumbricoides* and *T*. *trichiura* are thought to contribute to malnutrition, whereas hookworm and *T*. *trichiura* infections have been associated with iron-deficiency anaemia. [4, 5] All four main human STH species have been associated with impaired childhood growth. [5–7] Anaemia and malnutrition may have a negative long-term impact on an individual’s health and productivity, [6, 7] with resulting economic ramifications contributing to the cycle of poverty. [3] To reduce the impact of STH on communities, comprehensive control strategies are required. An impediment to implementing cost-effective control programs is a lack of accurate information detailing the geographic distribution of STH infections. [8] Since the transmission of infective STH relies upon favourable environmental conditions, environmental factors may be used to identify high risk areas. [9] Over the past two decades, geographic information systems (GIS) coupled with remotely sensed environmental data have been used to identify areas of high STH infection risk in several countries, [10–15] allowing governments to develop cost-effective targeted STH control strategies. [16].

Due to constrained resources, there has been little useful geographical information obtained about STH distributions in Timor-Leste in the decade following the restoration of independence in 2002, making it difficult to target interventions to the locations where they are needed the most. In 2005 the Timor-Leste Ministry of Health (MoH) in conjunction with the World Health Organization began a mass drug administration (MDA) program to control STH, however this program ceased in 2008 due to funding shortages. [17] Since then there has been limited distribution of albendazole to children presenting at mobile health units that attempt to provide regular health care to remote populations. In 2012, a national cross-sectional study found 29.0% of children in grades 3–5 were infected with STH, ranging from 4% in the Manatuto district to 55% in the Dili district. [17] High STH levels in 2012, following years of intermittent control strategies, suggest the MoH programs have been inadequate in controlling STH. [17] In 2013, the MoH developed an integrated national plan targeting neglected tropical diseases, including a seven year MDA program aimed at reducing STH infections and eliminating lymphatic filariasis, due to begin in 2014. [18] However, limited resources have delayed program implementation, with the MoH re-starting the program in 2015 in a limited number of districts. [19] Given the limited resources available in Timor-Leste, cost-effective methods to identify populations at high risk of STH infections are needed.

In 2012, the ‘WASH for Worms’ (W4W) randomized controlled trial was initiated in the Manufahi district, Timor-Leste, aiming to evaluate the effectiveness of a community-based water, sanitation and hygiene (WASH) programme in reducing parasitic infections above gains achieved through mass chemotherapy alone. [19] In the context of the W4W trial, cross-sectional surveys were conducted at baseline on 24 villages, involving the collection of stool samples to assess parasitic infection status, as well as demographic data and geographic coordinates for each participating household. [19] Using this cross-sectional data, this study aimed to describe and predict the distribution of STH infection in Manufahi district using environmental variables. This study specifically focused on *A*. *lumbricoides* and *N*. *americanus*, as analysis has shown these species of STH to be most prevalent in the study area. [20].

## Methods

### Ethics statement

As part of the baseline cross-sectional surveys, informed written consent from study participants aged ≥18 years and parents/guardians of children <18 years was obtained. Additionally, informed written assent was sought from children 12–17 years old. Informed consenting illiterate participants provided an ink thumb print *in lieu* of a signature. [19] Ethics approval for the W4W RCT was granted from: The Australian National University Human Ethics Committee (protocol: 2014/311), The Timorese Ministry of Health Research and Ethics Committee (reference: 2011/51), and The University of Queensland Human Research Ethics Committee (project number: 2011000734). The W4W trial is registered with the Australian and New Zealand Clinical Trials Registry (ACTRN12614000680662).

### Study area and data collection

Manufahi is one of 13 districts in Timor-Leste. The district is rural and largely agrarian, with farmers predominantly using traditional small scale subsistence farming methods. [21, 22] The climate is tropical, with a relatively cool dry season between July–November and a humid wet season between December–May (these seasons reportedly vary). [23] Based on WorldClim data from 1950−2000, [24] the average annual precipitation for Manufahi was approximately 1900 mm, whilst the average annual temperature was 24.5°C. The terrain is highly variable, ranging from flat coastal plains in the south composed of predominantly clay soil, to mountain ranges in the north with predominantly sandy loam and clay soils. [25] Villages involved in the cross-sectional baseline survey of the W4W trial were selected using detailed criteria. [19] Specifically, all selected villages were rural, had low access to WASH, and a willingness to participate in the W4W trial. [19].

All residents from the selected villages were eligible to participate in the cross-sectional survey provided they were present, greater than or equal to one year old, and they or a parent or guardian (if under 18) provided informed consent. [19] Baseline parasitological surveys were undertaken in the enrolled villages prior to any interventions, and were staggered over an 18 month period between May 2012 and October 2013. [19] At baseline, standardised questionnaires were administered to each participant inquiring about demographic factors and questions pertaining to WASH practices. [19] Additionally, standardised household and village questionnaires were administered to collect household and village characteristics, with global positioning system devices (Garmin handheld GPS, Garmin Ltd) used to determine the exact geographical position of each household. [19] Each participant was asked to provide one faecal specimen, which was separated into 2–3 ml aliquots and preserved in 15 ml centrifuge tubes containing 6ml of potassium dichromate (5%) for molecular analysis conducted at the QIMR Berghofer Medical Research Institute. Multiplex real-time polymerase chain reaction (qPCR) was used to identify and quantify the following STH infections: *Ascaris* spp., *N*. *americanus*, *Ancylostoma* spp., and *Trichuris trichiura*. [26] Given the W4W trial had molecular evidence that the vast majority of *Ascaris* spp. infections were indeed *A*. *lumbricoides* infections, [27] *Ascaris* spp. infections will herein be referred to as *A*. *lumbricoides* infections.

### Inclusion criteria

All eligible participants in the W4W cross-sectional baseline surveys were included in this analysis where age, sex and infection status were available. Of the 2,827 residents present and eligible to participate in the baseline cross-sectional survey, 99.1% (2,802/2,827) consented, and 78.5% (2,219/2,827) had infection status determined using qPCR (due to not all participants providing stool). Of those with infection status determined (2,219), sex status was missing for 0.99% (22/2,219), and age for 0.1% (3/2,219). After restricting the sample, 2,194 people in 606 households were in the study population for the analysis presented here.

### Obtaining and processing environmental data

The following environmental data were obtained for analysis: temperature, precipitation, elevation, soil texture and pH, landcover and vegetation (Table 1). All environmental variable processing was conducted in the GIS ArcMap 10.3 (ESRI, Redlands, CA), unless otherwise specified.

**Table 1:** Summary of environmental data sources considered in this analysis.

Environmental data type	Source	Temporal resolution	Spatial resolution (m)
Ambient temperature	WorldClim†	Monthly average from 1950–2000	1000
Precipitation	WorldClim†	Monthly average from 1950–2000	1000
Elevation	ASTER GDEM‡	2001	30
Vegetation (NDVI,EVI)	MODIS Terra satellite¥	01/01/2012-31/01/2013	250
Soil pH/ texture	Os Solos De Timor survey#	1960’s	N/A
Landcover	MODIS Terra and Aqua satellites*	2012	500

† WorldClim Version 1.4 (release 3)

‡ ASTER GDEM Version 2

¥ MOD13Q1, Version 5

# Os Solos De Timor data available from Seeds of Life Timor

* MCD12Q1, Version 5.1. ASTER GDEM: Advanced Spaceborne Thermal Emission and Reflection Radiometer Global Digital Elevation Model.

Long-term average annual and seasonal temperature (average, maximum and minimum) and precipitation variables were created using data retrieved from WorldClim at 1 km spatial resolution. [24] These layers were produced by using a thin-plate smoothing spline algorithm to interpolate data collected from global weather station sources between 1950 − 2000, [24] and have been validated for Timor-Leste by Molyneux et al. [28] Seasonality (e.g. wettest quarter) was assigned based upon the WorldClim monthly averages for Manufahi. [24].

Average vegetation indices (normalized difference vegetation index (NDVI) and enhanced vegetation index (EVI)), landcover, elevation and slope variables were created using data courtesy of the NASA EOSDIS Land Processes Distributed Active Archive Center (LP DAAC; see https://lpdaac.usgs.gov/). 16 day NDVI/EVI (MOD13Q1) data were obtained from January 1st 2012 to December 31st 2013, whilst yearly composite landcover (MCD12Q1) data were only available for 2012. [29, 30] Prior to developing long-term average variables for vegetation and landcover, the MODIS Reprojection Tool (version 4.1) [31] was used to convert data into a usable projection system for use in ArcGIS, and pixels underwent quality assessment. Landcover classes were defined using the International Geosphere Biosphere Programme (IGBP) classification system, which provides 11 natural vegetation classes, three non-vegetated classes, and three urban/agricultural classes. [32] The Advanced Spaceborne Thermal Emission and Reflection Radiometer (ASTER) global digital elevation model (GDEM) data at 30m spatial resolution were used, [33] and smoothed using Sun’s denoising algorithm. [34] This smoothed data were also used to determine the slope (in degrees).

Soil pH and texture variables were created based upon data from the 1960’s ‘Os Solos De Timor’ study, which sampled soil layers from over 285 locations across Timor-Leste. [35] Soil data were accessed from the Seeds of Life Timor website (http://seedsoflifetimor.org/). [25] Soil pH was classified using the United States Department of Agriculture (USDA) classification system. [36].

To be used in the univariate and multivariate analysis described below, all environmental variables were obtained in a 1 km radius around each household, and land attributes (soil texture, pH and landcover) also obtained for each household point (HHP). One kilometer buffer variables for EVI, NDVI, temperature, rainfall, elevation and slope were determined based on the median value within a 1 km radius of each household. The median value was thought to provide a more accurate and representative measure of exposure over an area thought to represent the average range of movement of participants from the HHP. [37] For land attributes, each household was assigned the land attribute category covering the majority of area within a 1 km radius. However, in some villages the land attributes differed markedly across a 1 km radius. Therefore, to account for potential micro-climates around the household, categorical land attribute variables were also obtained for each HHP. When determining soil texture and pH for each HHP, data were missing for some households in Lalmamir, Datina, Sarin and Ahiklatun villages. In these cases, the soil texture and pH were assigned based on the geographically closest soil type.

### Statistical analysis

All analysis was conducted using the R Statistical Software, version 3.2.0. [38] Some variables were transformed to improve model stability and interpretation of results, as outlined below. Elevation was expressed per 100 m, precipitation in centimetres, and EVI/NDVI in 0.01unit increments. As the categorical variable, soil texture, had several categories, some of which had a low sample size, univariable multilevel mixed-effects logistic regression with random-effect terms at the village and household levels was run for this variable against each outcome. As loam and clay loam categories had very similar odds ratios and it made sense to combine them based on composition, they were combined. This produced a soil texture variable with five groups: ‘clay/clay loam’, ‘loam’, ‘sandy clay’, ‘sandy loam’ and ‘variable’ soil type.

To prevent highly collinear variables from being in the same model, variables were grouped into domains within which many variables were collinear. To determine domain groupings, the Pearson’s correlation coefficient (*r*) for each pair of continuous variables was assessed, and separate domains created for each categorical variable. Specifically, these domains were: elevation/temperature, rainfall/slope, EVI/NDVI, landcover, soil type and soil pH, sex and age.

Univariable mixed-effects logistic regression with random effects at the village and household level was conducted for each independent variable. Additionally, for continuous independent variables, univariable models including orthogonal quadratic transformations of continuous independent variables were also considered, to allow for the possibility of a non-linear relationship between each continuous environmental variable and the probability of infection. Multivariable multilevel mixed-effects regression with random-effect terms at the village and household levels accounting for age and sex were developed by including the independent variable from each domain with the lowest Akaike Information Criterion (AIC). Two variables from separate domains were not allowed to enter the same model if the pair’s *r* was greater than 0.90. To create the final model for each helminth, working models were simplified for parsimony by dichotomising categorical variables if this reduced the AIC, followed by removing variables with p > 0.25 on the Wald test.

To determine if the final models adequately accounted for spatial autocorrelation, semivariograms were created based on the village random intercepts for each given model. Semivariograms describe how data are related with respect to distance and direction, with a semivariogram presenting the similarity between observations (semivariance) at different separation distances. [39] Omni-directional semivariograms based on the village random intercepts were developed in R using the geoR package.[40] Lack of distinctive spatial patterns in these semivariograms (S1 Fig) supported that the final models adequately accounted for spatial clustering at the village level.

### Development of risk maps

Separate predictive risk maps for *A*. *lumbricoides* and *N*. *americanus* in the highest risk age and sex groups were created based upon the parameter estimates of the various environmental and demographic variables included in the final model for each species. For the *A*. *lumbricoides* model, the coefficient for children aged 6-<18 years was included, and for the *N*. *americanus* model, the coefficient for adults (≥18 year old) was included. For display purposes (i.e., map visualization) smoothing was used. In this context, missing predictions arising from missing soil data (texture and pH) were completed by allocating the mean value of the neighboring cells in a 2 by 2 pixel area.

To develop the risk map for any STH in 6-<18 year olds, separate predictive risk maps for *A*. *lumbricoides* and *N*. *americanus* were developed as described above; however, the models used to create these maps incorporated the coefficient for children aged 6-<18 years, and separate risk maps were generated for both males and females (adding the coefficient for females in the latter). Missing predictions were handled in the same manner as described above. For each sex, the predicted risk maps for *A*. *lumbricoides* and *N*. *americanus* in 6–18 year olds was then summed, and the product of the risk maps subtracted in order to address coinfections. This manner of addressing coinfections assumes that individuals infected with one species were no more or less likely to be infected with the second species than uninfected individuals. The predicted risk maps for any STH in males and females were then averaged, assuming equal distribution of both sexes, to produce an overall predicted risk map of any STH in 6 to 18 year olds. Predictions were then categorized based on the World Health Organisation (WHO) thresholds for deworming school aged children, namely low risk (<20%), moderate risk (≥20-<50%) and high risk (≥50%) [41].

## Results

### Descriptive statistics

**Fig 1:**
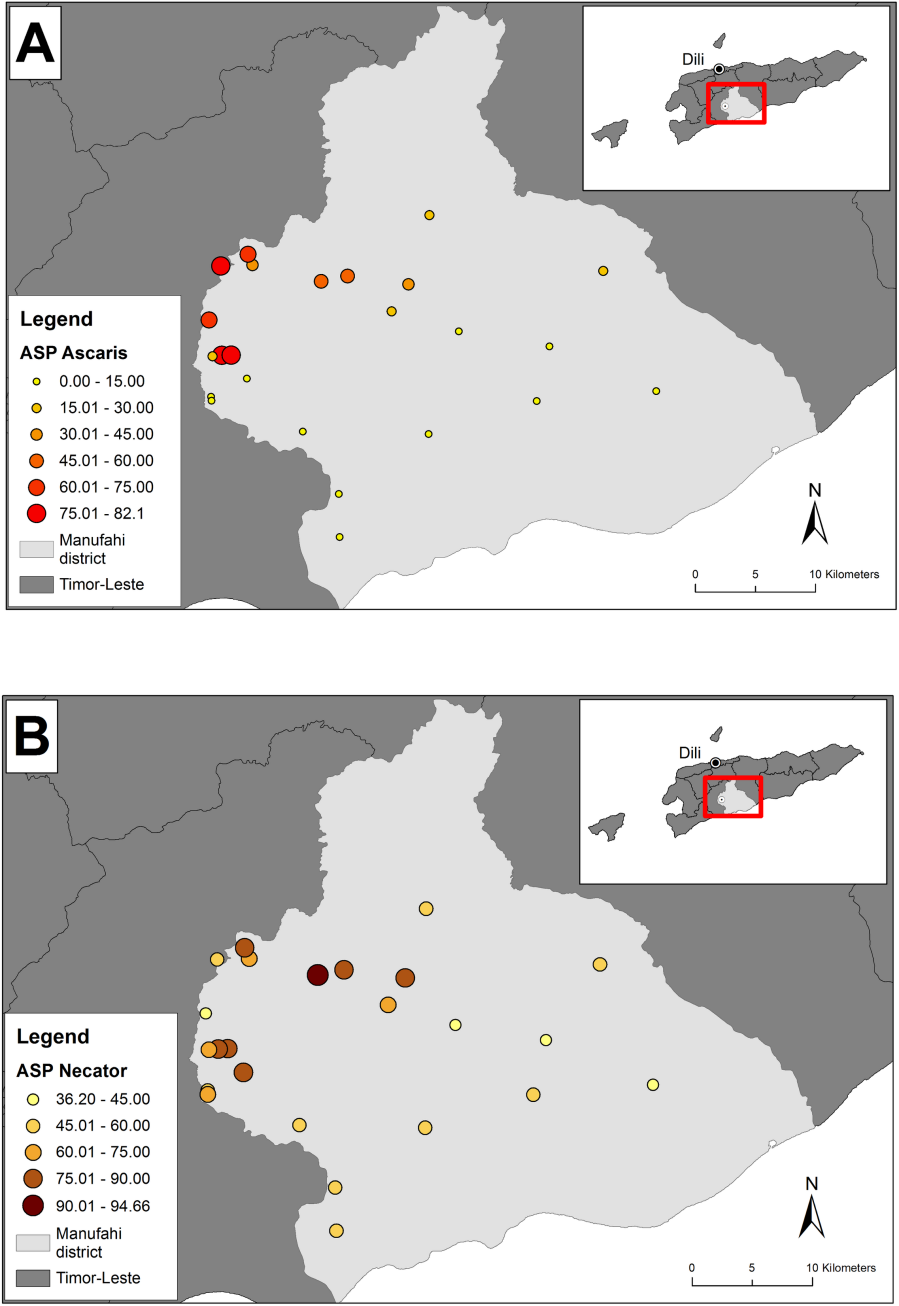
Baseline age standardised prevalence (ASP) of (A) *A*. *lumbricoides* and (B) *N*. *americanus* in 24 villages in Manufahi district, Timor-Leste. ***A*. *lumbricoides* has been shortened to *Ascaris*, and *N*. *americanus* to *Necator***.

The study population (n = 2,194) was 51.3% female, and ranged in age from 1–92 years (median 19.0 years) (Table 2). The number of participants varied between villages, ranging from 29–318 (mean 91.4 participants). Overall, the unadjusted prevalence for *A*. *lumbricoides* was 24.2% (ranging from 0.0–79.6% between villages) and 60.7% for *N*. *americanus* (ranging from 39.2–94.5% between villages). Since the age structure varied across the villages, the age standardised prevalence (ASP) of *A*. *lumbricoides* and *N*. *americanus* was determined for each village. *A*. *lumbricoides* ASP was heterogeneous across villages, ranging from 0% –82.0% (Fig 1A). *N*. *americanus* ASP was higher overall, ranging from 36.2–94.7% (Fig 1B).

**Table 2:** Characteristics of environmental conditions surrounding study participants' households.

Variable	Median/ n(%)	IQR	Minimum	Maximum
**Environmental variables**				
Annual mean temperature (°C)‡	24.5	3.5	16.5	26.7
Annual maximum temperature (°C)‡	28.4	3.8	20.3	30.7
Annual minimum temperature (°C)‡	20.7	3.3	12.7	22.7
Mean temperature in hottest quarter (°C)‡	25.7	3.6	17.5	27.8
Mean temperature in coldest quarter (°C)‡	23	3.7	14.8	25.3
Maximum temperature in hottest month (°C)‡	30	3.6	22.1	32.1
Minimum temperature in coldest month (°C)‡	18.6	3.3	11	20.6
Annual mean precipitation (cm)‡	17.1	5	12.9	20.4
Mean precipitation in driest quarter (cm)‡	2.9	1.4	1.8	4.4
Mean precipitation in wettest quarter (cm)‡	29.1	12.1	21.6	37.5
Precipitation in driest month (cm)‡	1.7	0.6	1.2	2.5
Precipitation in wettest month (cm)‡	30.5	9	24.2	36.5
Elevation (m)‡	419.2	604.91	33.99	1469
Slope (°)‡	14.1	14.8	1.7	25.2
EVI [–1,1]‡	0.46	0.05	0.38	0.53
NDVI [–1,1]‡	0.75	0.07	0.64	0.80
**Soil pH¥**				
Moderately acidic	263 (12%)			
Slightly acidic	471 (21.5%)			
Neutral	1218 (55.5%)			
Slightly alkaline	101 (4.6%)			
Moderately alkaline	141 (6.4%)			
**Soil texture**				
Clay	503 (22.9%)			
Clay loam	19 (0.9%)			
Loam	669 (30.5%)			
Sandy clay	350 (16.0%)			
Sandy loam	565 (25.8%)			
Variable	88 (4.0%)			
**Landcover#**				
Cropland/natural vegetation mosaic	641 (29.2%)			
Evergreen forest	770 (35.1%)			
Savanna	104 (4.7%)			
Woody savanna	679 (30.9%)			
**Adjustment variables**				
Age (years)	19	34	1	92
Female	51.3			
Male	48.7			

n = 2,194, IQR: Interquartile Range

‡ Median value in 1km radius of household

¥ Category at HHP

# Category covering majority of area in 1 km radius of household.

### Univariable analysis

Results of univariable mixed-effects logistic regression analysis for *A*. *lumbricoides* and *N*. *americanus* are detailed in S1–S4 Tables. For *A*. *lumbricoides*, the environmental variable with the lowest AIC in each group was: mean temperature in the coldest quarter, average NDVI, slope, soil pH at HHP (three groups), soil texture at HHP and landcover at a 1 km buffer (S1 and S2 Tables). The AIC’s of univariable models with only the linear environmental term (S1 Table) did not meaningfully differ (< 4 points, guided by the rule of thumb reported by Burnham and Anderson [42]) to the respective AIC’s of models containing both the linear and the quadratic terms (S2 Table), and thus for parsimony, linear forms of continuous environmental variables were included in the working multivariable model. For *N*. *americanus*, the environmental variable with the lowest AIC in each group was: elevation (quadratic form), average EVI, mean precipitation in the driest month, predominant soil pH (three groups) and landcover in 1km buffer of household, and soil texture (five groups) at the HHP (S3 and S4 Tables). Although models including the quadratic forms of temperature and elevation lowered the AIC by approximately 10 points (S4 Table), they were unstable (large standard errors), and so the continuous and categorical forms of elevation were considered for model selection (S3 Table).

### Model selection

The full and final environmental multivariable models for *A*. *lumbricoides* and *N*. *americanus* infections are detailed in Tables 3 and 4 respectively. The AIC of the working *A*. *lumbricoides* model was improved by binarising soil texture and landcover variables by combining several categories, and removing NDVI. For *N*. *americanus*, the model was improved by binarising soil texture and removing soil pH and landcover. The continuous form of elevation was used in the final *N*. *americanus* model as this form of elevation resulted in the model with the lowest AIC.

**Table 3:** Multivariable associations between environmental and demographic factors and the likelihood of *A*. *lumbricoides* infection, using multilevel mixed-effects logistic regression (n = 2,194).

	Full model		Final model after variable elimination	
Covariates	OR (95% CI)	*p* value	OR (95% CI)	*p* value
(Intercept)	204.96 (0.02–2035972.17)	0.257	144.4 (0.64–32642.97)	0.072
Mean temperature in coldest quarter (per 1°C)	0.75 (0.60–0.93)	0.010	0.74 (0.60–0.92)	0.006
Slope (per 1°)	1.08 (0.97–1.19)	0.164	1.07 (0.98–1.16)	0.110
NDVI average (per 0.01)	0.99 (0.87–1.13)	0.898	-	-
Acidic soil	Reference		Reference	
Neutral soil	0.68 (0.37–1.25)	0.214	0.68 (0.37–1.23)	0.203
Alkaline soil	0.28 (0.08–0.98)	0.046	0.27 (0.08–0.96)	0.043
Clay loam/loam soil (compared to other)	0.23 (0.09–0.56)	0.001	0.22 (0.09–0.54)	*<* 0.001
Woody savanna	0.51 (0.27–0.94)	0.030	0.51 (0.28–0.93)	0.027
Female sex	0.98 (0.76–1.27)	0.889	0.98 (0.76–1.27)	0.878
1–<6 year olds	Reference		Reference	
6–*<*18 years	1.70 (1.15–2.53)	0.008	1.68 (1.13–2.49)	0.010
*≥*18 years	0.779 (0.53–1.14)	0.195	0.77 (0.53–1.12)	0.173
AIC	1732.4		1730.4	

**Table 4:** Multivariable associations between environmental and demographic factors and the likelihood of *N*. *americanus* infection, using multilevel mixed-effects logistic regression (n = 2,194).

	Full Model with consolidated variables		Final model after variable elimination	
Covariates	OR (95% CI)	p value	OR (95% CI)	p value
(Intercept)	0.00 (0.00–0.04)	< 0.001	0.00 (0.00–0.01)	< 0.001
Elevation (per 100 m)	0.91 (0.84–0.97)	0.006	0.91 (0.85–0.97)	0.004
Precipitation in driest month (cm)	7.41 (2.76–19.88)	< 0.001	6.54 (2.94–14.57)	< 0.001
EVI average (per 0.01)	1.06 (0.98–1.14)	0.164	1.07 (1.00–1.15)	0.040
Acidic soil	Reference		-	-
Neutral soil pH	1.06 (0.62–1.80)	0.839	–	–
Alkaline soil pH	0.78 (0.44–1.39)	0.405	–	–
Sandy loam	2.51 (1.55–4.07)	< 0.001	2.50 (1.56–4.01)	< 0.001
Croplands/natural vegetation mosaic	Reference		Reference	
Evergreen forest	0.98 (0.50–1.95)	0.964	–	–
Savanna	1.40 (0.70–2.81)	0.346	–	–
Woody savanna	0.91 (0.52–1.57)	0.728	–	–
Female sex	0.35 (0.28–0.43)	< 0.001	0.34 (0.27–0.43)	< 0.001
1-<6 years	Reference		Reference	
6–<18 years	5.32 (3.76–7.53)	< 0.001	4.98 (3.54–7.02)	< 0.001
≥18 years	9.97 (7.08–14.06)	< 0.001	9.37(6.68–13.16)	< 0.001
AIC	2484.0		2477.1	

For *A*. *lumbricoides*, the final model following variable elimination indicated temperature in the coldest quarter, slope, soil pH, soil texture, landcover and age were associated with the odds of infection in the study site (Table 3). Increasing temperatures in the coldest quarter of the year were associated with a decreased odds of infection (OR: 0.74, 95% Confidence Interval [CI]: 0.60–0.92, p = 0.006). Clay loam/loam soils around the household were associated with reduced infection odds compared to other soil types (OR: 0.22, 95% CI: 0.09–0.54, p < 0.001). Having predominantly woody savanna in a 1 km radius of households significantly lowered infection odds compared to other vegetation types (OR: 0.51, 95% CI: 0.28–0.93, p = 0.027). Alkaline soils were associated with lower odds of infection compared to acidic soils (OR: 0.27, 95%CI: 0.08–0.96, p = 0.043). After adjusting for environmental factors, a non-linear association between age and infection odds was evident, as 6–<18 year olds had significantly higher infection odds compared to 1–<6 year olds (OR: 1.68, 95% CI: 1.13–2.49, p = 0.010), and no significant difference between 6-<18 year olds and ≥18 year olds (OR: 0.77, 95% CI: 0.53–1.12, p = 0.173). The random effect terms included in the model (village, and household) reveal there was greater variation at the village level (variance: 0.77) than the household level (variance: 0.55).

For *N*. *americanus*, the final model, after variable elimination, suggests elevation, precipitation, average EVI, soil texture, sex and age were associated with infection (Table 4). Greater elevations were associated with reduced odds of infection (OR: 0.91, 95% CI: 0.85–0.97, p = 0.004), whilst higher precipitation in the driest month of the year significantly increased infection odds (OR: 6.54, 95% CI: 2.94–14.57, p < 0.001). Higher EVI values were associated with increased infection odds (OR: 1.07, 95% CI: 1.00–1.15, p = 0.040). Having predominantly sandy loam soils in a 1 km buffer of the household was associated with greater than double the odds of infection compared to other soil types (OR: 2.50, 95% CI: 1.56–4.01, p < 0.001). Sex was significantly associated with infection, with females having a significantly reduced odds of infection compared to males (OR: 0.34, 95% CI: 0.27–0.43, p < 0.001). In relation to age, compared to 1–<6 year olds, 6–<18 year olds had 4.98 times higher infection odds (95% CI: 3.54–7.02, p < 0.001), and adults had 9.37 times higher odds (95% CI: 6.68–13.16, p < 0.001). For the random effect terms, village and households, there was greater variation at the household level (variance = 0.69) than the village level (variance = 0.09).

### Risk maps for *A*. *lumbricoides* and *N*. *americanus*

**Fig 2:**
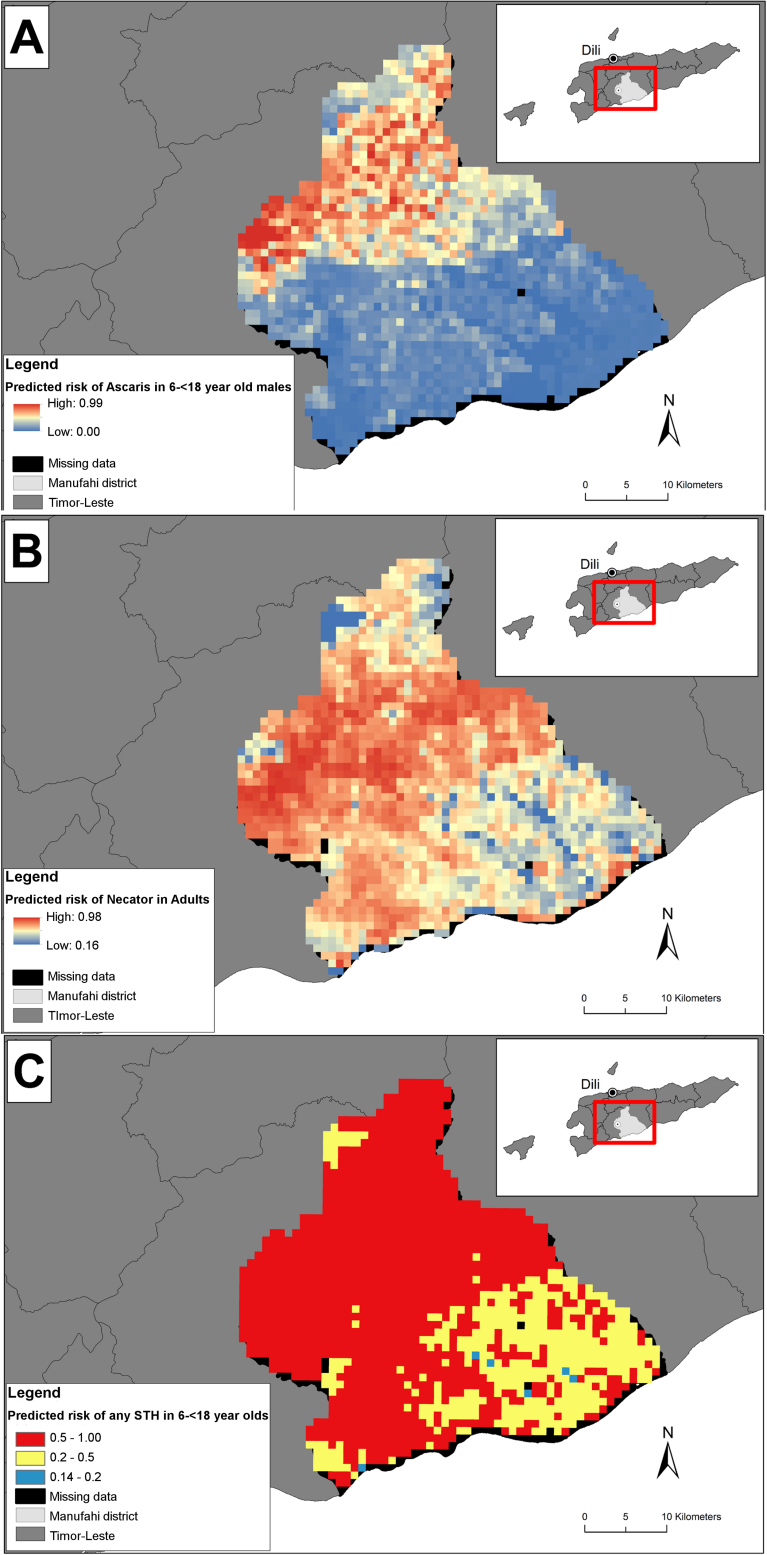
Predictive risk maps for *A*. *lumbricoides*,*N*. *americanus* and any STH infection in Manufahi. (A) Predicted risk of *A*. *lumbricoides* infection in the highest risk group, 6–<18 year old males, (B) Predicted risk of *N*. *americanus* infection in the highest risk group, ≥18 year old males, (C) Predicted risk of any STH infection in 6–<18 year olds, according to WHO school-age children infection thresholds for deworming. *A*. *lumbricoides* has been shortened to *Ascaris*, and *N*. *americanus* to *Necator*.

Fig 2A and 2B present the predicted risk of infection with *A*. *lumbricoides* and *N*. *americanus* in males in the highest risk age groups (6–<18 years and ≥18 years respectively). Note, risk predictions for other age and sex categories will have an identical spatial distribution, but with an overall lower mean. In Fig 2A, the predicted risk of infection with *A*. *lumbricoides* was higher in the central and northern parts of Manufahi, which have lower temperatures, more acidic soils and higher elevation and precipitation compared to the lower risk southern coastal regions. The sudden change from low to medium/high predicted risk is due to a combination of decreased temperatures and the soils in the region being more acidic and of variable soil types (predominantly sandy clay and sandy loam), all of which are associated with an increased odds of *A*. *lumbricoides* infection (Table 3). In Fig 2B, the predicted risk of *N*. *americanus* was high across the district, with the highest predicted risk concentrated in the central region. The coastal southeastern area had a lower risk, likely due to having higher temperatures, lower elevations, lower precipitation, and predominantly clay soils (Table 4). Some missing data are shown in the maps–these are locations where soil texture and pH data were unavailable, and where the infilling using the mean of the nearest 2 x 2 pixels was unable to provide an estimate due to there being no neighbouring pixels with prediction estimates.

Fig 2C depicts the predicted risk of any STH in 6-<18 year olds, based on WHO prevalence thresholds for deworming in school aged children. Fig 2C highlights that the majority of 6-<18 year olds in Manufahi have a high predicted risk of infection (≥50%), with the remainder having moderate predicted risk (≥20 to <50%).

## Discussion

Our results support that *A*. *lumbricoides* and *N*. *americanus* infections favoured different environmental conditions in the study area, influencing the predicted geographical distribution of human infection with these species in Manufahi district.

Loam and clay loam soils were associated with lower odds of *A*. *lumbricoides* infection whilst sandy loam soils were associated with increased odds of *N*. *americanus* infection, compared to other soil types. The finding that sandy loam soils were favourable for *N*. *americanus* is biologically plausible [9] and in accordance with a number of epidemiological studies. [43–45] The porous environment of sand provides a favourable environment for hookworm survival, offering drainage during wet conditions to prevent hookworm larvae from being waterlogged, whilst also enabling hookworm larvae to migrate downwards during hot and dry conditions to prevent dessication. [9] The finding that loam and clay loam soils were negatively associated with *A*. *lumbricoides* infection is in contrast to the available literature which suggests clay and loam soils are more favourable for *A*. *lumbricoides* egg development compared to sandy soils. [46, 47] This was an unanticipated finding, but one explanation could be residual confounding, such as other soil properties, exposure to ultraviolet radiation, and human interaction. [8, 9].

Contrasting relationships with temperature/elevation were observed for *A*. *lumbricoides* and *N*. *americanus*, suggesting that different species favour different climatic conditions. Higher temperatures in the coldest quarter of the year were associated with reduced odds of *A*. *lumbricoides* infection, suggesting *A*. *lumbricoides* favours cooler conditions in tropical areas. This supports epidemiological and biological evidence that *A*. *lumbricoides* is prone to desiccation in hot conditions.[9, 48] In contrast, the odds of infection with *N*. *americanus* were higher at lower elevations that have slightly warmer temperatures, potentially reflecting the ability of hookworm to survive in warmer temperatures due to their motility.[9] This negative relationship has been well documented by studies across Africa.[49–52].

Having predominantly woody savanna in a 1 km radius of households was unfavourable for *A*. *lumbricoides* infection compared to other vegetation types assessed. Woody savannas have an understorey plant system, and a forest canopy that covers between 30–60% of the environment. [53] This reasonably high level of vegetation, shade, and thus moisture, should in theory be conducive to *A*. *lumbricoides* survival and development. [9] This finding may therefore reflect micro-climates (e.g. soil properties), human movement patterns, or possibly livelihoods and related occupational exposures, which may potentially reduce transmission in areas with woody savanna.

Alkaline soils were associated with reduced odds of *A*. *lumbricoides* infection, suggesting *A*. *lumbricoides* favours acidic conditions. This finding is in agreement with Chammartin et al. who found acidic soils ranging between pH 5.35 and 5.65 were associated with increased odds of *A*. *lumbricoides* infection [11]. Although not evident in this study, Chammartin et. al also found a negative association between pH and the risk of hookworm infection, [11] which is supported by a laboratory study that reported that N. *americanus* favours acidic conditions. [54] Combined, these studies provide evidence to support further research into using products that reduce soil acidity as part of interventions to control STH, namely lime, which is used routinely in agriculture to increase productivity. [55, 56].

Greater precipitation in the driest month of the year was associated with increased odds of *N*. *americanus* infection, supporting studies that find hookworm requires a minimum level of soil moisture or humidity throughout the year to survive and develop. [9, 15, 57] The positive association between EVI and *N*. *americanus* infection is consistent with Saathoff et al. [44] As EVI is a measure of vegetation and thus a proxy for environmental moisture and shade, this finding further supports that hookworm require sufficient soil moisture for survival and transmission. [9, 54].

The age trends identified in this study were consistent with the available literature and earlier analysis. [20] School-aged children (6–<18 years) had a greater odds of *A*. *lumbricoides* infection compared to pre-school aged children (1–<6 years) and adults (≥18 years). [37, 58–60] On the other hand, the odds of *N*. *americanus* infection increased with age. [1, 7, 58, 61, 62] Males were found to have a higher odds of *N*. *americanus* infection, whilst no association was evident for *A*. *lumbricoides*. The higher odds of hookworm infection in males may reflect occupational roles or behavioural factors that could increase exposure of males to infection. [63].

The predictive risk maps highlight that 6 to 18 year olds were at high to medium risk of having a STH infection in a large portion of Manufahi. Given that STH infections may have immediate and long term health and economic impacts, [3] the widespread predicted risk of STH in Manufahi provides evidence to support that a district wide STH control strategy is needed. Ideally any such strategy would include regular deworming with antihelminthic drugs such as albendazole or mebendazole integrated with WASH interventions. [64] Importantly, studies suggest co-infected individuals are at a higher risk of helminth related morbidity. [65, 66] This is thought to be because of the morbidity associated with each species, [5–7] and because co-infected individuals generally have heavier infections of each worm species. [65–69] Our maps suggest that the central area of Manufahi is likely to have a higher presence of co-infections.

The study has several strengths. Since the W4W trial is a randomised controlled trial, individual and household data were collected in a systematic manner using trial protocols, [19] reducing the likelihood of information bias. Importantly, misclassification was minimized in this study, as the W4W trial employed the use of qPCR to detect STH infections, which is a more sensitive technique compared to standard microscopy.[26] The use of qPCR and including entire communities (as opposed to school-age children only) may explain the higher infection levels reported here, compared to the 2012 national survey. As household GPS coordinates were available, the highest resolution open-access environmental data available could be used to determine individual exposures as accurately as possible, minimising measurement error and improving the accuracy of environmental parameter estimates. Our analysis included soil pH and soil texture data, which are often not available for spatial studies. We found both soil pH and soil texture variables were associated with the likelihood of STH infection, supporting the wider use of these variables in developing explanatory and predictive models for STH. Future studies should therefore endeavour to collect information on soil texture, pH and other properties (e.g. ions, carbon to nitrogen ratio etc.) to better define risk and potentially offer additional strategies for STH control.

Our analysis however did have some limitations. The soil data used were collected in the 1960s and alongside missing data it may be inaccurate at the time of the study. Socio-demographic and behavioural factors were not included in the analysis as data were unavailable or outdated for all of Manufahi, meaning risk maps could not incorporate these variables. A separate analysis of behavioural and sociodemographic factors in the W4W trial provides detailed information on these factors. [20] Moreover, the W4W trial did not utilise a spatial sampling frame, as spatial analysis was not the main purpose of the trial. A spatial sampling frame would have been more efficient and provided a more uniform coverage of the study area. Furthermore, the “smoothed” maps were produced making the following assumptions: to address coinfections, we assumed that individuals infected with one species were as likely to be infected with a second species as uninfected individuals, and to produce the overall predicted risk map of any STH in 6–<18 year olds, we assumed equal distribution of both sexes in each prediction location. If different species of STH cluster in the same individual we might have overestimated the total prevalence of infection. Additionally, the actual prevalence in each location might be affected by an unequal distribution of the sexes but given that we did not have data on sex distributions in non-sampled locations we were unable to incorporate this in the predictions. However, we believe that neither issue was likely to change significantly the conclusions of the study. It should also be noted that the risk maps are based on the environmental and demographic variables included in the model. There will be other factors that influence the distribution of disease risk. Some of these factors vary at the village and household levels, and some of this variance would have been captured in the random effects. These maps therefore show the relative difference in risk of each location based on their environmental characteristics, adjusted for any confounding related to age and sex, and robust to clustering of the data at the village level. For the scale of the analysis (a single district of Timor-Leste) it is reasonable to assume the relationship between disease risk and the environmental characteristics is uniform across the region. Lastly, “smoothing” of missing values for soil texture and pH data may limit the accurate representation of risk in the small number of locations where these data were missing.

In conclusion, in the Manufahi district, STH infection is strongly associated with several environmental factors. The widespread predicted risk of any STH infection across the study area provides good evidence that control strategies are required for the entire geographical area. Our research combined with other epidemiological and biological studies suggests that products that increase soil pH, such as liming, could be investigated as a potential avenue for control.

## Supporting information

S1 Checklist STROBE checklist. (DOCX)

S1 Dataset Village dataset. (PDF)

S1 Codebook Village codebook.
(PDF)

S1 Fig Omnidirectional semivariograms based on the residuals from multivariable mixed effects logistic regression models for (A) *A*. *lumbricoides* and (B) *N*. *americanus*.
(TIF)

S1 Table Univariable associations between linear or categorical environmental and demographic variables and the likelihood of *A*. *lumbricoides* infection, using multilevel mixed effects logistic regression (n = 2,194). (DOCX)

S2 Table Univariable multilevel mixed-effects logistic regression beta coefficients for quadratic forms of continuous environmental variables and the likelihood of *A*. *lumbricoides* infection (n = 2,194). (DOCX)

S3 Table Univariable associations between linear or categorical environmental and demographic variables and *N*. *americanus* infection, using multilevel mixed-effects logistic regression (n = 2,194). (DOCX)

S4 Table Univariable multilevel mixed-effects logistic regression beta-coefficients for quadratic forms of continuous environmental variables and the likelihood of *N*. *americanus* infection (n = 2,194). (DOCX)
