# Supplementary material for: An environmental assessment and risk map of Ascaris lumbricoides and Necator americanus distributions in Manufahi District, Timor-Leste
Source: PLoS Negl Trop Dis. 2017 May 10;11(5):e0005565. doi: 10.1371/journal.pntd.0005565 (PMC5440046; doi:10.1371/journal.pntd.0005565)
Supplement: S1 Dataset — (PDF) [file pntd.0005565.s002.pdf]

Village_nu	Village	Intervention_village	Mean_latitude	Mean_longitude
1	Foelora	1	-9.002718	125.6162
2	Dare-Ba	0	-9.002924	125.6091
3	Datina	1	-8.934946	125.6318
4	Lia Nai	1	-9.0203	125.6282
5	Lesuhati	0	-8.935804	125.6078
6	Falitehu	0	-8.946758	125.6838
7	Batas	0	-8.942703	125.7037
8	Manggaet	1	-8.92681	125.6284
9	Grotu 2	1	-9.034146	125.6011
10	Grotu 3	1	-9.03718	125.6015
11	Sarin	1	-8.994401	125.8567
12	Hatu-Hei	1	-8.976401	125.5993
13	Ahi-Klatun	0	-8.983653	125.7881
14	Besakrem	0	-9.003676	125.6021
15	Luak	0	-9.059781	125.6708
16	Kakao Oan	0	-9.035528	125.8474
17	Bandeira	0	-9.06096	125.7659
18	Lalmamir	1	-9.106479	125.6983
19	Raifusa	0	-9.13887	125.6992
20	Fatu-Cilat	1	-8.896428	125.7652
21	Morbady	1	-8.948624	125.7498
22	ıata-Asin-Be	0	-8.969011	125.7372
23	Quirantetue	1	-8.937377	125.8969
24	Tatekar	0	-9.02736	125.9377

Median_elevation	Median_age	Sample_population	N_with_Ascaris	Crd_pw_Ascaris
730.4344	9	49	39	79.59184
710.1686	11	29	23	79.31034
1013.789	22	100	42	42
649.9422	22.5	58	7	12.06897
1405.525	13	67	52	77.61194
413.7404	15.5	54	26	48.14815
827.053	22	55	28	50.90909
1220.564	17	65	47	72.30769
523.9171	30	78	5	6.410256
515.0256	24	90	6	6.666667
178.3983	13	127	0	0
1190.508	22	57	35	61.40351
263.033	25.5	78	9	11.53846
710.618	28	45	9	20
198.9105	27	53	7	13.20755
128.0909	19.5	88	4	4.545455
119.5688	22.5	100	4	4
105.565	26	48	0	0
31.20914	18	313	12	3.833866
1125.768	20.5	114	30	26.31579
772.6611	13	194	85	43.81443
715.1565	17	95	24	25.26316
343.1498	21	117	29	24.78632
87.76688	17	75	3	4

Adj_pw_Ascaris	N_with_necator	Crd_pw_Necator	Adj_pw_Necator	b1km_astelev_inm
82.07713	41	83.67347	84.83017	724.57
77.68789	22	75.86207	76.91976	753.23
42.77596	65	65	64.04841	1075.31
12.32232	50	86.2069	87.56925	636.29
76.30836	32	47.76119	51.32494	1450.48
48.14395	51	94.44444	94.51062	508.99
49.77096	49	89.09091	85.86952	760.38
73.49524	47	72.30769	75.43341	1168.63
6.151467	46	58.97436	59.78752	432.52
6.913469	57	63.33333	62.30207	411.9
0	48	37.79528	38.93664	181.25
61.66375	25	43.85965	42.38824	1245.34
12.43959	30	38.46154	35.75743	271.76
19.34489	28	62.22222	60.20082	738.66
14.80446	31	58.49057	54.00674	228.37
4.243807	48	54.54545	51.05566	132.99
3.727539	54	54	51.73732	138.36
0	23	47.91667	45.91223	108.45
3.6908	179	57.1885	56.61662	45.67
26.37009	69	60.52632	59.14971	1054.13
42.55943	148	76.28866	76.68253	656.74
25.35623	65	68.42105	68.15094	601.5
25.21741	59	50.42735	50.60306	370.18
4.032808	30	40	39.99576	85.65

b1km_ast_slope	b1km_pre_mean_yrdiv10	b1km_pre_3dry_div10	b1km_pre_3wet_div10
17.39	20.3	3.9	35.6
18.37	20.4	4.2	36.2
18.36	18.9	3.6	36.7
15.58	20.3	3.6	34.7
18.9	18.4	3.4	36.5
21.41	18.1	2.9	30.5
20.81	19.8	3.8	35.7
23.18	18.8	3.6	36.7
17.53	17.8	3	30.3
18.34	17.8	3	30.3
3.11	14.3	2	23.8
15.55	18.7	3.6	36.8
4.82	15.1	2.2	25.3
17.13	20.4	4.2	36.2
7.3	14.8	2.4	24.9
2.51	13.9	2	23.1
6.08	13.6	2	22.7
3.03	13.4	2.1	22.5
3.25	12.8	1.9	21.5
18.52	18.4	3	35.3
18.42	19.7	3.3	33.9
14.12	19.2	3.1	32.3
9.01	15.9	2.2	26.6
1.96	13.5	1.8	22.1

b1km_pre_1wet_div10	b1km_pre_9dry_div10	b1km_tmean_yr	b1km_tmean_3hot
35.7	2.4	23.1	24.2
36.2	2.5	22.7	23.8
34.8	1.9	20.5	21.4
35	2.3	23.3	24.5
34.1	1.8	18.7	19.6
31.3	2	24.1	25.2
35.1	2.2	22.3	23.3
34.7	1.9	20.5	21.4
31.6	2.1	24.5	25.7
31.6	2.1	24.5	25.7
25.9	1.4	25.8	26.9
34.7	1.9	19.8	20.8
27.1	1.6	25.5	26.6
36.2	2.5	22.7	23.8
26.9	1.7	25.7	26.9
25.2	1.4	26	27.2
24.9	1.5	26.3	27.4
24.9	1.5	26.3	27.4
24.1	1.4	26.7	27.8
33.2	1.7	20.8	21.7
34	2.1	23	24.1
32.9	2	23.2	24.4
28.5	1.4	24.7	25.8
24.4	1.2	26.3	27.4

b1km_tmean_3cold	b1km_tmax_mean_yr	b1km_tmin_yr	b1km_tmax_novhot
21.4	26.9	19.3	28.5
21	26.5	19	28.2
19.1	24.3	16.8	25.8
21.6	27.1	19.5	28.8
17.2	22.5	15	24
22.5	28	20.3	29.6
20.7	26.2	18.6	27.8
19	24.2	16.8	25.7
23	28.4	20.7	30
23	28.4	20.7	30
24.4	30	21.8	31.4
18.3	23.6	16.1	25.2
24	29.6	21.6	31
21	26.5	18.9	28.1
24.3	29.7	21.8	31.2
24.6	30.1	22	31.6
24.8	30.3	22.3	31.7
24.8	30.3	22.3	31.6
25.3	30.7	22.7	32.1
19.3	24.6	17	26.1
21.4	26.9	19.2	28.5
21.6	27.1	19.4	28.8
23.2	28.8	20.7	30.4
24.9	30.5	22.2	31.9

b1km_tmin_augcold	b1km_range_11tmax8tmin	b1km_NDVI	b1km_EVI	ph_1kmb
17.1	11.4	0.7815	0.4825	6.3
16.8	11.4	0.7778	0.4738	6.3
15	10.8	0.7713	0.4752	5.9
17.3	11.4	0.787	0.5241	7.8
13.2	10.9	0.6886	0.3972	6.3
18.2	11.4	0.7633	0.463	6
16.5	11.3	0.7824	0.4941	6.3
15	10.8	0.7506	0.4551	5.9
18.6	11.4	0.7673	0.4911	7.8
18.6	11.4	0.7639	0.484	7.8
19.7	11.6	0.6484	0.3888	6.8
14.3	10.9	0.7789	0.464	6.6
19.5	11.5	0.7484	0.4629	7.3
16.8	11.3	0.7513	0.4668	6.3
19.7	11.5	0.6955	0.4223	7
20	11.6	0.7043	0.4181	7.5
20.2	11.5	0.6884	0.4256	7
20.2	11.4	0.7261	0.4494	7.5
20.6	11.4	0.724	0.4537	7
15.2	10.9	0.791	0.4903	6.6
17.1	11.4	0.7887	0.5103	6.3
17.3	11.5	0.7703	0.4837	6
18.6	11.7	0.7237	0.462	7
20.1	11.8	0.6737	0.422	7.9

ph_5_1km_grp	ph_3_1km_grp	texture_1kmb	texture_1km_5grp
Slightly acidic	Acidic	Sandy_Clay	Sandy Clay
Slightly acidic	Acidic	Sandy_Clay	Sandy Clay
Moderately acidic	Acidic	Sandy_Loam	Sandy Loam
Slightly alkaline	Alkaline	Clay	Clay
Slightly acidic	Acidic	Sandy_Loam	Sandy Loam
Moderately acidic	Acidic	Sandy_Loam	Sandy Loam
Slightly acidic	Acidic	Sandy_Clay	Sandy Clay
Moderately acidic	Acidic	Sandy_Loam	Sandy Loam
Slightly alkaline	Alkaline	Clay	Clay
Slightly alkaline	Alkaline	Clay	Clay
Neutral	Neutral	Clay	Clay
Neutral	Neutral	Sandy_Loam	Sandy Loam
Neutral	Neutral	Clay	Clay
Slightly acidic	Acidic	Sandy_Clay	Sandy Clay
Neutral	Neutral	Clay	Clay
Slightly alkaline	Alkaline	Loam	Clay Loam/Loam
Neutral	Neutral	Clay	Clay
Slightly alkaline	Alkaline	Clay	Clay
Neutral	Neutral	Clay_Loam	Clay Loam/Loam
Neutral	Neutral	Sandy_Loam	Sandy Loam
Slightly acidic	Acidic	Sandy_Clay	Sandy Clay
Moderately acidic	Acidic	Sandy_Loam	Sandy Loam
Neutral	Neutral	Clay	Clay
Moderately alkaline	Alkaline	Loam	Clay Loam/Loam

landcover_assign_1km
Evergreen forest
Evergreen forest
Woody Savanna
Evergreen forest
Cropland/Natural Veg
Evergreen forest
Evergreen forest
Woody Savanna
Evergreen forest
Evergreen forest
Woody Savanna
Evergreen forest
Woody Savanna
Evergreen forest
Cropland/Natural Veg
Cropland/Natural Veg
Cropland/Natural Veg
Cropland/Natural Veg
Cropland/Natural Veg
Evergreen forest
Woody Savanna
Evergreen forest
Woody Savanna
Cropland/Natural Veg
