# Supplementary material for: An environmental assessment and risk map of Ascaris lumbricoides and Necator americanus distributions in Manufahi District, Timor-Leste
Source: PLoS Negl Trop Dis. 2017 May 10;11(5):e0005565. doi: 10.1371/journal.pntd.0005565 (PMC5440046; doi:10.1371/journal.pntd.0005565)
Supplement: S1 Codebook — Village codebook. (PDF) [file pntd.0005565.s003.pdf]

Variable used	Variable name in dataset	Units	Village point or 1 km buffer?
Village number	Village_nu	Integer	N/A
Village name	Village	N/A	N/A
Mean latitude of village	Mean_latitude	Decimal degrees	point
Mean longitude of village	Mean_longitude	Decimal degrees	point
Median elevation for registered households in villages	Median_elevation	Meters	N/A
Median age of village members	Median_age	Years	N/A
Number of people in village	Sample_population	Persons	N/A
Number of people in village with ascaris	N_with_Ascaris	Persons	N/A
Crude percentage of people in village with ascaris	Crd_pw_Ascaris	Percentage (%)	N/A
Age standardized prevalence of ascaris in village	Adj_pw_Ascaris	Percentage (%)	N/A
Number of people in village with necator	N_with_necator	Persons	N/A
Crude percentage of people in village with necator	Crd_pw_Necator	Percentage (%)	N/A
Age standardized prevalence of necator in village	Adj_pw_Necator	Percentage (%)	N/A
Elevation (m)	b1km_astelev_inm	Meters	median value in 1 km buffer of village GPS coordinates
Slope (°)	b1km_ast_slope	Degrees	median value in 1 km buffer of village GPS coordinates
Monthly average precipitation (cm)	b1km_pre_mean_yrdiv10	cm	median value in 1 km buffer of village GPS coordinates
Mean precipitation in driest quarter (August, September, October) (cm)	b1km_pre_3dry_div10	cm	median value in 1 km buffer of village GPS coordinates
Mean precipitation in wettest quarter (December, January, February) (cm)	b1km_pre_3wet_div10	cm	median value in 1 km buffer of village GPS coordinates
Precipitation in wettest month (January) (cm)	b1km_pre_1wet_div10	cm	median value in 1 km buffer of village GPS coordinates
Precipitation in driest month (September) (cm)	b1km_pre_9dry_div10	cm	median value in 1 km buffer of village GPS coordinates
Annual mean temperature (°C)	b1km_tmean_yr	°C	median value in 1 km buffer of village GPS coordinates
Mean temperature in warmest quarter (October, November December) (°C)	b1km_tmean_3hot	°C	median value in 1 km buffer of village GPS coordinates
Mean temperature in coldest quarter (June, July, August) (°C)	b1km_tmean_3cold	°C	median value in 1 km buffer of village GPS coordinates
Annual maximum temperature(°C)	b1km_tmax_mean_yr	°C	median value in 1 km buffer of village GPS coordinates
Annual minimum temperature(°C)	b1km_tmin_yr	°C	median value in 1 km buffer of village GPS coordinates
Maximum temperature in hottest month (November) (°C)	b1km_tmax_novhot	°C	median value in 1 km buffer of village GPS coordinates

Minimum temperature in coldest month (August) (°C)	b1km_tmin_augcold	°C	median value in 1 km buffer of village GPS coordinates
Temperature range (Maximum temperature in the hottest month - Minimum temperature in coldest month) (°C)	b1km_range_11tmax8tmin	°C	median value in 1 km buffer of village GPS coordinates
Average NDVI	b1km_NDVI		median value in 1 km buffer of village GPS coordinates
Average EVI	b1km_EVI		median value in 1 km buffer of village GPS coordinates
Soil pH	ph_1kmb	pH scale	Covers greatest % of area in a 1km buffer of village GPS coordinates
Soil pH in five categories	ph_5_1km_grp	Categorical: Moderately Acidic, Slightly Acidic, Neutral, Slightly Alkaline, Moderately Alkaline	Covers greatest % of area in a 1km buffer of village GPS coordinates
Soil pH in three categories	ph_3_1km_grp	Categorical: Acidic, Neutral, Alkaline	Covers greatest % of area in a 1km buffer of village GPS coordinates
Soil texture	texture_1kmb	Categorical	Covers greatest % of area in a 1km buffer of village GPS coordinates
Soil texture 5 groups	texture_1km_5grp	Categorical: 5 groups: Clay, Clay Loam/Loam, Sandy Clay, Sandy Loam, Variable	Covers greatest % of area in a 1km buffer of village GPS coordinates
Landcover	landcover_assign_1km	Categorical: Cropland/Natural Veg , Evergreen forest, Savanna, Woody Savanna	Covers greatest % of area in a 1km buffer of village GPS coordinates
