# Supplementary figures and images for: An environmental assessment and risk map of Ascaris lumbricoides and Necator americanus distributions in Manufahi District, Timor-Leste
Source: PLoS Negl Trop Dis. 2017 May 10;11(5):e0005565. doi: 10.1371/journal.pntd.0005565 (PMC5440046; doi:10.1371/journal.pntd.0005565)

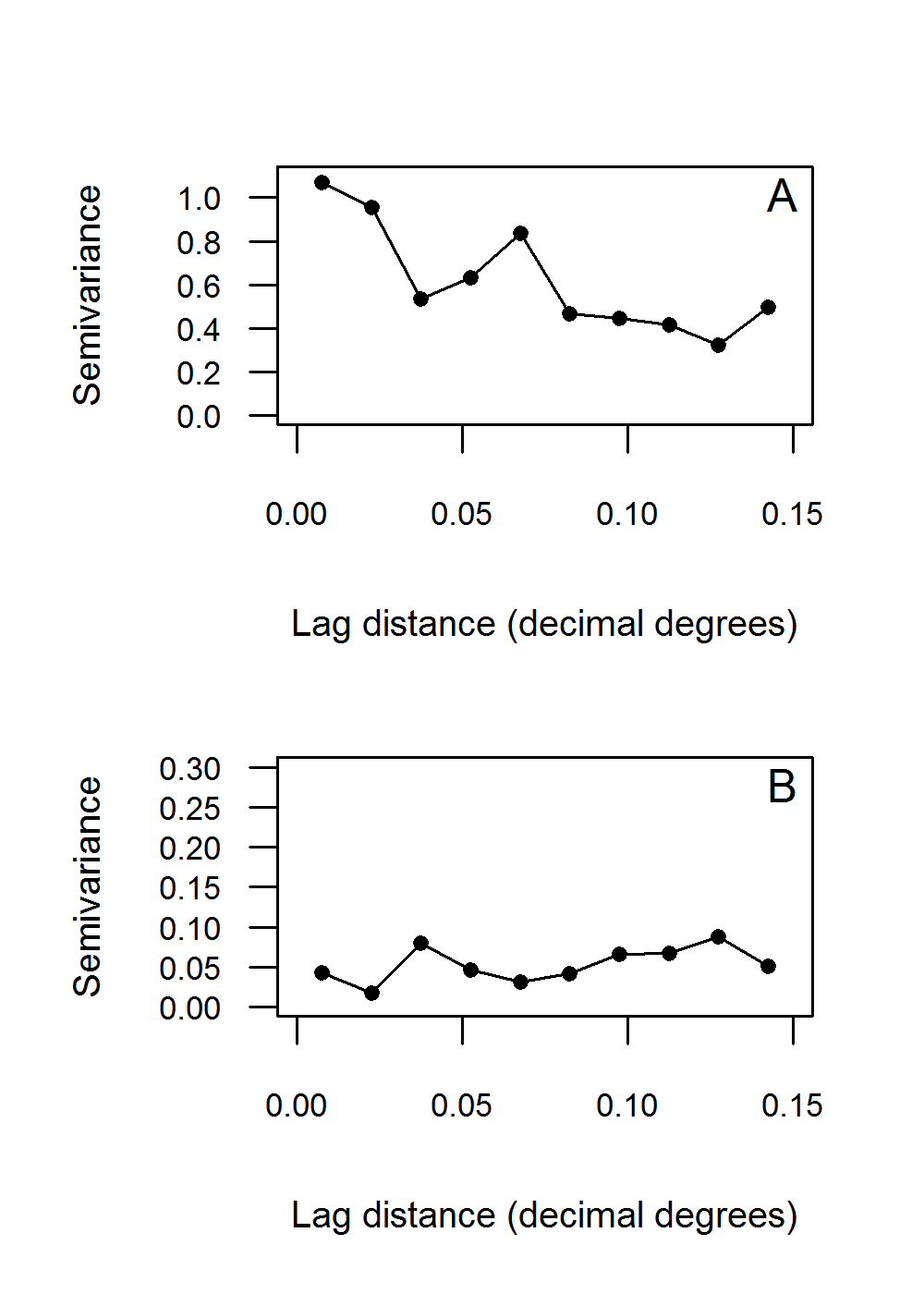

Supplement: S1 Fig — Omnidirectional semivariograms based on the residuals from multivariable mixed effects logistic regression models for (A) A. lumbricoides and (B) N. americanus. (TIF) [file pntd.0005565.s004.tif]
