# Supplementary material for: An environmental assessment and risk map of Ascaris lumbricoides and Necator americanus distributions in Manufahi District, Timor-Leste
Source: PLoS Negl Trop Dis. 2017 May 10;11(5):e0005565. doi: 10.1371/journal.pntd.0005565 (PMC5440046; doi:10.1371/journal.pntd.0005565)
Supplement: S1 Table — (DOCX) [file pntd.0005565.s005.docx]

S1 Table:

| **Domain** | **Variable** | **OR (95% CI)** | **p value** | **AIC** |
| --- | --- | --- | --- | --- |
| **Temperature/ Elevation** | Elevation (per 100m) | 1.49 (1.49–1.49) | < 0.001 | 1770.63 |
|  | Annual mean temperature (◦C) | 0.55 (0.55–0.55) | < 0.001 | 1771.26 |
|  | Annual maximum temperature (◦C) | 0.56 (0.48–0.67) | < 0.001 | 1771.30 |
|  | Annual minimum temperature (◦C) | 0.54 (0.54–0.54) | < 0.001 | 1772.27 |
|  | Mean temperature in hottest quarter (◦C) | 0.56 (0.55–0.56) | < 0.001 | 1771.19 |
|  | Mean temperature in coldest quarter (◦C) | 0.55 (0.47–0.66) | < 0.001 | 1770.42 |
|  | Maximum temperature in hottest month (◦C) | 0.56 (0.47–0.67) | < 0.001 | 1772.03 |
|  | Minimum temperature in coldest month (◦C) | 0.52 (0.43–0.63) | < 0.001 | 1771.14 |
|  | Temperature range | 0.02 (0.00 –0.15) | < 0.001 | 1784.02 |
| **Precipitation/ slope** | Slope (°) | 1.23 (1.15–1.32) | < 0.001 | 1773.50 |
|  | Annual mean precipitation (cm) | 1.72 (171–1.72) | < 0.001 | 1782.69 |
|  | Mean precipitation in driest quarter (cm) | 5.41 (5.40–5.42) | < 0.001 | 1782.57 |
|  | Mean precipitation in wettest quarter (cm)† | 1.31 (1.22–1.42) | < 0.001 | 1772.40 |
|  | Precipitation in driest month (cm) | 4.93 (0.78–31.03) | 0.089 | 1796.65 |
|  | Precipitation in wettest month (cm) | 1.42 (1.27–1.59) | < 0.001 | 1775.89 |
| **Vegetation** | NDVI average (per 0.01) | 1.17 (1.03–1.33) | 0.019 | 1794.24 |
|  | EVI average (per 0.01) | 1.02 (0.85–1.21) | 0.851 | 1798.91 |
| **Soil pH** | **Soil pH three categories HHP** |  |  |  |
|  | Acidic | Reference |  |  |
|  | Neutral | 0.35 (0.19–0.67) | 0.001 | 1785.12 |
|  | Alkaline | 0.09 (0.02–0.43) | 0.003 |  |
|  | **Soil pH three categories 1km radius** |  |  |  |
|  | Acidic | Reference |  |  |
|  | Neutral | 0.53 (0.53–0.54) | < 0.001 | 1787.52 |
|  | Alkaline | 0.06 (0.06–0.06) | < 0.001 |  |
|  | **Soil pH ﬁve categories HHP** |  |  |  |
|  | Moderately acidic | Reference |  |  |
|  | Slightly acidic | 1.18 (1.18–1.18) | < 0.001 | 1787.44 |
|  | Neutral | 0.41 (0.41–0.41) | < 0.001 |  |
|  | Slightly alkaline | 0.15(0.15–0.15) | < 0.001 |  |
|  | Moderately alkaline | 0.04 (0.04–0.04) | < 0.001 |  |
|  | **Soil pH ﬁve categories 1 km radius** |  |  |  |
|  | Moderately acidic | Reference |  |  |
|  | Slightly acidic | 2.16 (2.15–2.16) | < 0.001 | 1787.71 |
|  | Neutral | 0.95 (0.95– 0.95) | < 0.001 |  |
|  | Slightly alkaline | 0.10 (0.10–0.10) | < 0.001 |  |
|  | Moderately alkaline | 0.08 (0.08–0.08) | < 0.001 |  |
| **Soil texture** | **Soil texture ﬁve categories HHP** |  |  |  |
|  | Clay | Reference |  |  |
|  | Clay loam/loam | 0.09 (0.03–0.27) | < 0.001 | 1774.82 |
|  | Sandy clay | 1.78 (0.79–4.02) | 0.167 |  |
|  | Sandy loam | 0.65 (0.29–1.42) | 0.275 |  |
|  | Variable | 0.08 (0.00–23.1) | 0.174 |  |
|  | **Soil texture ﬁve categories 1 km radius** |  |  |  |
|  | Clay | Reference |  |  |
|  | Clay loam/loam | 0.42 (0.11–1.66) | 0.217 | 1792.98 |
|  | Sandy clay | 2.15 (0.79–5.89) | 0.135 |  |
|  | Sandy loam | 0.72 (0.28–1.85) | 0.500 |  |
|  | Variable | 0.00 (0.00–2.37^22^) | 0.630 |  |
|  | **Soil texture all categories HHP** |  |  |  |
|  | Clay | Reference |  |  |
|  | Clay loam | 0.00 (0.00–1.48^19^) | 0.578 | 1775.63 |
|  | Loam | 0.09 (0.03–0.28) | < 0.001 |  |
|  | Sandy clay | 1.78 (0.79–4.02) | 0.167 |  |
|  | Sandy loam | 0.65(0.30–1.42) | 0.278 |  |
|  | Variable | 0.08 (0.00–3.06) | 0.174 |  |
|  | **Soil texture all categories 1 km radius** |  |  |  |
|  | Clay | Reference |  |  |
|  | Clay loam | 0.44 (0.10–1.97) | 0.284 | 1794.96 |
|  | Loam | 0.40 (0.09–1.83) | 0.238 |  |
|  | Sandy clay | 2.15 (0.79–5.88) | 0.135 |  |
|  | Sandy loam | 0.72 (0.28–1.85) | 0.500 |  |
|  | Variable | 0.00 (0.00–1.24^75^) | 0.863 |  |
| **Landcover** | **Landcover HHP** |  |  |  |
|  | Croplands/natural vegetation mosaic | Reference |  |  |
|  | Evergreen forest | 1.58 (0.67–3.73) | 0.297 | 1799.98 |
|  | Savanna | 1.21 (0.39 – 3.80) | 0.740 |  |
|  | Woody savanna | 0.97 (0.39-2.41) | 0.947 |  |
|  | **Landcover 1 km radius** |  |  |  |
|  | Croplands/natural vegetation mosaic | Reference |  |  |
|  | Evergreen forest | 2.72 (0.67–11.14) | 0.163 | 1793.49 |
|  | Savanna | 0.37 (0.09 –1.57) | 0.177 |  |
|  | Woody savanna | 1.15 (0.25–5.15) | 0.851 |  |
| **Potential confounders** | **Age categorical** |  |  |  |
|  | 1–<6 years | Reference |  |  |
|  | 6–<18 years | 1.58 (1.08 –2.31) | 0.019 | 1774.48 |
|  | ≥18 years | 0.75 (0.52 –1.08) | 0.116 |  |
|  | Continuous form of age (per year) | 0.98 (0.98–0.99) | < 0.001 | 1773.31 |
|  | Female | 0.96 (0.75–1.22) | 0.720 | 1798.82 |

† Mean precipitation in wettest quarter could not be included in working model despite having the lowest AIC in the precipitation/slope domain because it was highly collinear with variables in temperature/elevation domain.
