# Supplementary material for: An environmental assessment and risk map of Ascaris lumbricoides and Necator americanus distributions in Manufahi District, Timor-Leste
Source: PLoS Negl Trop Dis. 2017 May 10;11(5):e0005565. doi: 10.1371/journal.pntd.0005565 (PMC5440046; doi:10.1371/journal.pntd.0005565)
Supplement: S2 Table — (DOCX) [file pntd.0005565.s006.docx]

S2 Table

| **Domains** | **Covariates** | **β (95% CI)** | **p value** | **AIC** |
| --- | --- | --- | --- | --- |
| Temperature/ elevation | Elevation (per 100m) | 77.99 (56.52 – 99.46) | < 0.001 | 1770.41 |
|  | Quadratic | -15.31 (-34.56 – 3.93) | 0.119 |  |
|  | Annual mean temperature (◦C) | -75.33 (-95.16 – -55.51) | < 0.001 | 1767.50 |
|  | Quadratic | -22.34 (-40.30 – -4.38) | 0.015 |  |
|  | Annual maximum temperature (◦C) | -74.92 (-95.78– -54.06) | <0.001 | 1768.22 |
|  | Quadratic | -20.78 (-38.84 – -2.72) | 0.024 |  |
|  | Annual minimum temperature (◦C) | -74.29(-93.47 – -55.10) | <0.001 | 1768.30 |
|  | Quadratic | -22.77 (-39.88 – -5.66) | 0.009 |  |
|  | Mean temperature in hottest quarter (◦C) | -74.90 (-94.69 – -55.12) | <0.001 | 1767.35 |
|  | Quadratic | -22.62 (-40.51 – -4.73) | 0.013 |  |
|  | Mean temperature in coldest quarter (◦C) | -76.03 (-96.39 – -55.68) | <0.001 | 1767.08 |
|  | Quadratic | -21.23 (-39.50 – -2.97) | 0.023 |  |
|  | Maximum temperature in hottest month (◦C) | -73.60 (-93.45 – -53.75) | <0.001 | 1768.96 |
|  | Quadratic | -21.11 (-39.04 – -3.18) | 0.021 |  |
|  | Minimum temperature in coldest month (◦C) | -75.62 (-95.40 – -55.84) | <0.001 | 1767.22 |
|  | Quadratic | -22.40(-40.38 – -4.43) | 0.015 |  |
|  | Temperature range (◦C) | -50.52 (-72.59 – -28.45) | <0.001 | 1785.17 |
|  | Quadratic | -9.19 (-28.55 – 10.17) | 0.352 |  |
| Precipitation/ slope | Slope (◦) | 67.44 (43.33 – 91.56) | <0.001 | 1774.45 |
|  | Quadratic | 7.28 (-6.37 – 20.92) | 0.296 |  |
|  | Annual mean precipitation (cm) | 69.73 (44.09 – 95.37) | <0.001 | 1784.08 |
|  | Quadratic | -7.43 (-25.73 – 10.87) | 0.426 |  |
|  | Mean precipitation in driest quarter (cm) | 66.49 (42.65 – 90.32) | <0.001 | 1781.41 |
|  | Quadratic | -15.98 (-33.56 – 1.60) | 0.075 |  |
|  | Mean precipitation in wettest quarter (cm) | 71.82 (49.49 – 94.15) | <0.001 | 1774.28 |
|  | Quadratic | 3.15 (-14.69 – 20.99) | 0.729 |  |
|  | Precipitation in driest month (cm) | 42.38(16.59 – 68.17) | 0.001 | 1794.14 |
|  | Quadratic | -23.74 (-43.85 – -3.62) | 0.021 |  |
|  | Precipitation in wettest month (cm) | 69.40 (46.32 – 92.48) | <0.001 | 1777.85 |
|  | Quadratic | 1.70 (-15.07 – 18.47) | 0.842 |  |
| Vegetation | NDVI average | 20.58 (-7.85 – 49.02) | 0.156 | 1793.31 |
|  | Quadratic | 16.91 (-107 – 34.89) | 0.065 |  |
|  | EVI average | 18.42 (-12.47 – 49.31) | 0.242 | 1796.34 |
|  | Quadratic | -25.22 (-47.84 – -2.61) | 0.029 |  |

Note: Quadratic refers to the second order polynomial term
