# Supplementary material for: An environmental assessment and risk map of Ascaris lumbricoides and Necator americanus distributions in Manufahi District, Timor-Leste
Source: PLoS Negl Trop Dis. 2017 May 10;11(5):e0005565. doi: 10.1371/journal.pntd.0005565 (PMC5440046; doi:10.1371/journal.pntd.0005565)
Supplement: S3 Table — (DOCX) [file pntd.0005565.s007.docx]

S3 Table:

| **Domain** | **Variable** | **OR (95% CI)** | **p value** | **AIC** |
| --- | --- | --- | --- | --- |
| **Temperature/ Elevation** | Elevation (per 100m) | 1.06 (0.98 – 1.14) | 0.164 | 2786.08 |
|  | Annual mean temperature (◦C) | 0.93 (0.83 – 1.05) | 0.259 | 2786.69 |
|  | Annual maximum temperature (◦C) | 0.93 (0.83 – 1.04) | 0.223 | 2786.50 |
|  | Annual minimum temperature (◦C) | 0.93 (0.82 – 1.06) | 0.323 | 2786.97 |
|  | Mean temperature in hottest quarter (◦C) | 0.94 (0.83 – 1.05) | 0.301 | 2786.89 |
|  | Mean temperature in coldest quarter (◦C) | 0.92 (0.82 – 1.04) | 0.218 | 2786.46 |
|  | Maximum temperature in hottest month (◦C) | 0.93 (0.82 – 1.05) | 0.258 | 2786.68 |
|  | Minimum temperature in coldest month (◦C) | 0.93 (0.81 – 1.06) | 0.281 | 2786.80 |
|  | Temperature range | 0.51 (0.18 – 1.42) | 0.214 | 2786.43 |
| **Precipitation/ slope** | Slope (°) | 1.07 (1.04 – 1.11) | < 0.001 | 2775.22 |
|  | Annual mean precipitation (cm) | 1.24 (1.13 – 1.36) | < 0.001 | 2773.35 |
|  | Mean precipitation in driest quarter (cm) | 1.87 (1.35 – 2.60) | < 0.001 | 2778.06 |
|  | Mean precipitation in wettest quarter (cm) | 1.08 (1.03 – 1.13) | 0.003 | 2780.87 |
|  | Precipitation in driest month (cm) | 5.10 (2.79 – 9.30) | < 0.001 | 2769.70 |
|  | Precipitation in wettest month (cm) | 1.12 (1.05 –1.19) | < 0.001 | 2777.93 |
| **Vegetation** | NDVI average (per 0.01) | 1.12 (1.07 – 1.18) | < 0.001 | 2774.39 |
|  | EVI average (per 0.01) | 1.16 (1.09 – 1.24) | < 0.001 | 2772.99 |
| **Soil pH** | **Soil pH three categories HHP** |  |  |  |
|  | Acidic | Reference |  |  |
|  | Neutral | 1.13(0.61 – 2.11) | 0.699 | 2789.71 |
|  | Alkaline | 0.98 (0.36 – 2.64) | 0.960 |  |
|  | **Soil pH three categories 1km radius** |  |  |  |
|  | Acidic | Reference |  |  |
|  | Neutral | 1.32 (0.73 – 2.36) | 0.357 | 2786.86 |
|  | Alkaline | 0.77 (0.37 – 1.59) | 0.482 |  |
|  | **Soil pH ﬁve categories HHP** |  |  |  |
|  | Moderately acidic | Reference |  |  |
|  | Slightly acidic | 0.53 (0.26 – 1.08) | 0.082 | 2789.21 |
|  | Neutral | 0.61 (0.26 – 1.41) | 0.251 |  |
|  | Slightly alkaline | 0.70 (0.22 – 2.23) | 0.545 |  |
|  | Moderately alkaline | 0.36 (0.10 – 1.22) | 0.100 |  |
|  | **Soil pH ﬁve categories 1 km radius** |  |  |  |
|  | Moderately acidic | Reference |  |  |
|  | Slightly acidic | 0.48 (0.25 – 0.95) | 0.036 | 2786.29 |
|  | Neutral | 0.73 (0.34 – 1.56) | 0.412 |  |
|  | Slightly alkaline | 0.49 (0.21 – 1.18) | 0.112 |  |
|  | Moderately alkaline | 0.33 (0.09 – 1.24) | 0.099 |  |
| **Soil texture** | **Soil texture ﬁve categories HHP** |  |  |  |
|  | Clay | Reference |  |  |
|  | Clay loam/loam | 1.13 (0.61 – 2.12) | 0.694 | 2781.60 |
|  | Sandy clay | 1.48 (0.75 – 2.93) | 0.259 |  |
|  | Sandy loam | 2.69 (1.49 – 4.85) | 0.001 |  |
|  | Variable | 0.96 (0.23 – 4.06) | 0.955 |  |
|  | **Soil texture ﬁve categories 1 km radius** |  |  |  |
|  | Clay | Reference |  |  |
|  | Clay loam/loam | 0.61 (0.29 – 1.27) | 0.184 | 2781.94 |
|  | Sandy clay | 1.41 (0.74 – 2.67) | 0.298 |  |
|  | Sandy loam | 2.25 (1.28 – 3.99) | 0.005 |  |
|  | Variable | 0.47 (0.09 – 2.44) | 0.368 |  |
|  | **Soil texture all categories HHP** |  |  |  |
|  | Clay | Reference |  |  |
|  | Clay loam | 1.49 (0.41 – 5.44) | 0.550 | 2783.38 |
|  | Loam | 1.11 (0.59 – 2.09) | 0.735 |  |
|  | Sandy clay | 1.49 (0.75 – 2.94) | 0.255 |  |
|  | Sandy loam | 2.68 (1.49 – 4.84) | 0.001 |  |
|  | Variable | 0.96 (0.23 – 4.04) | 0.952 |  |
|  | **Soil texture all categories 1 km radius** |  |  |  |
|  | Clay | Reference |  |  |
|  | Clay loam | 0.76 (0.33 – 1.74) | 0.511 | 2782.78 |
|  | Loam | 0.53 (0.24 – 1.15) | 0.109 |  |
|  | Sandy clay | 1.39 (0.73 – 2.64) | 0.313 |  |
|  | Sandy loam | 2.23 (1.26 – 3.93) | 0.006 |  |
|  | Variable | 0.51 (0.10 – 2.65) | 0.421 |  |
| **Landcover** | **Landcover HHP** |  |  |  |
|  | Croplands/natural Vegetation mosaic | Reference |  |  |
|  | Evergreen forest | 1.94 (1.16 – 3.24) | 0.011 | 2785.14 |
|  | Savanna | 1.10 (0.60 – 2.00) | 0.758 |  |
|  | Woody savanna | 1.14 (0.68 – 1.91) | 0.624 |  |
|  | **Landcover 1 km radius** |  |  |  |
|  | Croplands/natural vegetation mosaic | Reference |  |  |
|  | Evergreen forest | 2.48(1.37 – 4.52) | 0.003 | 2782.69 |
|  | Savanna | 1.67 (0.85 – 3.09) | 0.142 |  |
|  | Woody savanna | 1.77 (0.91 – 3.44) | 0.095 |  |
| **Potential confounders** | **Age categorical** |  |  |  |
|  | 1–<6 years | Reference |  |  |
|  | 6–<18 years | 4.64 (4.63 – 4.65) | < 0.001 | 2604.4 |
|  | ≥18 years | 8.19 (8.17 –8.20) | < 0.001 |  |
|  | Continuous form of age (per year) | 1.03 (1.03 –1.03) | < 0.001 | 2630.65 |
|  | Female | 0.41 (0.34 – 0.51) | < 0.001 | 2712.9 |
