# Supplementary material for: An environmental assessment and risk map of Ascaris lumbricoides and Necator americanus distributions in Manufahi District, Timor-Leste
Source: PLoS Negl Trop Dis. 2017 May 10;11(5):e0005565. doi: 10.1371/journal.pntd.0005565 (PMC5440046; doi:10.1371/journal.pntd.0005565)
Supplement: S4 Table — (DOCX) [file pntd.0005565.s008.docx]

S4 Table:

| **Domains** | **Covariates** | **β (95% CI)** | **p value** | **AIC** |
| --- | --- | --- | --- | --- |
| Temperature/ elevation | Elevation (per 100m) | 15.37 (4.73 – 26.01) | 0.005 | 2772.36 |
|  | Quadratic | -23.94(-34.53 – -13.34) | <0.001 |  |
|  | Annual mean temperature (◦C) | -12.61 (-23.79 – -1.43) | 0.027 | 2776.02 |
|  | Quadratic | -21.61 (-32.31 – -10.92) | <0.001 |  |
|  | Annual maximum temperature (◦C) | -13.07 (-23.84 – -2.30) | 0.017 | 2775.16 |
|  | Quadratic | -21.80 (-32.14 – -11.45) | <0.001 |  |
|  | Annual minimum temperature (◦C) | -11.58 (-23.03 – -0.12) | 0.048 | 2777.6 |
|  | Quadratic | -20.84 (-32.00 – -9.68) | <0.001 |  |
|  | Mean temperature in hottest quarter (◦C) | -11.94 (-23.05 – -0.83) | 0.035 | 2776.6 |
|  | Quadratic | -21.46 (-32.21 – -10.71) | <0.001 |  |
|  | Mean temperature in coldest quarter (◦C) | -13.31 (-24.38 – -2.24) | 0.018 | 2775.62 |
|  | Quadratic | -21.56 (-32.33 – -10.79) | <0.001 |  |
|  | Maximum temperature in hottest month (◦C) | -12.44 (-23.14 – -1.74) | 0.023 | 2774.52 |
|  | Quadratic | -22.37 (-32.73 – -12.01) | <0.001 |  |
|  | Minimum temperature in coldest month (◦C) | -12.36 (-23.73 – -0.99) | 0.033 | 2776.93 |
|  | Quadratic | -21.08 (-31.93 – -10.23) | <0.001 |  |
|  | Temperature range (◦C) | -7.81 (-19.39 – 3.77) | 0.186 | 2780.91 |
|  | Quadratic | -16.58 (-27.84 – -5.32) | 0.004 |  |
| Precipitation/ slope | Slope (◦) | 24.15 (12.85 – 35.45) | <0.001 | 2776.05 |
|  | Quadratic | -4.91 (-13.94 – 4.11) | 0.286 |  |
|  | Annual mean precipitation (cm) | 24.15 (13.62 – 34.68) | <0.001 | 2773.69 |
|  | Quadratic | 6.67 (-3.03 – 16.37) | 0.178 |  |
|  | Mean precipitation in driest quarter (cm) | 22.76 (11.62 – 33.89) | <0.001 | 2777.97 |
|  | Quadratic | -7.31 (-17.06 – 2.44) | 0.142 |  |
|  | Mean precipitation in wettest quarter (cm) | 21.92 (9.40 – 34.44) | <0.001 | 2778.87 |
|  | Quadratic | -11.73 (-23.06 – -0.41) | 0.042 |  |
|  | Precipitation in driest month (cm) | 25.84 (16.23 – 35.44) | <0.001 | 2771.58 |
|  | Quadratic | -1.53 (-10.14 – 7.07) | 0.727 |  |
|  | Precipitation in wettest month (cm) | 22.52 (10.39 – 34.66) | <0.001 | 2779.62 |
|  | Quadratic | -2.95 (-13.30 – 7.39) | 0.576 |  |
| Vegetation | NDVI average | 23.35 (12.54 – 34.16) | <0.001 | 2776.07 |
|  | Quadratic | -3.04 (-13.80 – 7.72) | 0.580 |  |
|  | EVI average | 24.25 (13.72 – 34.78) | <0.001 | 2774.41 |
|  | Quadratic | 4.11 (-6.22 – 14.44) | 0.436 |  |

Note: Quadratic refers to the second order polynomial term
